# Supplementary material for: Cytosine-5 RNA Methylation Regulates Neural Stem Cell Differentiation and Motility
Source: Stem Cell Reports. 2016 Dec 29;8(1):112–24. doi: 10.1016/j.stemcr.2016.11.014 (PMC5233436; doi:10.1016/j.stemcr.2016.11.014)
Supplement: Document S1. Figures S1 and S2 [file mmc1.pdf]

Stem Cell Reports, Volume *8*

Supplemental Information

Cytosine-5 RNA Methylation Regulates Neural Stem Cell Differentiation and Motility

Joana V. Flores, Lucía Cordero-Espinoza, Feride Oeztuerk-Winder, Amanda Andersson-Rolf, Tommaso Selmi, Sandra Blanco, Jignesh Tailor, Sabine Dietmann, and Michaela Frye

Figure S1, Related to Figure 2

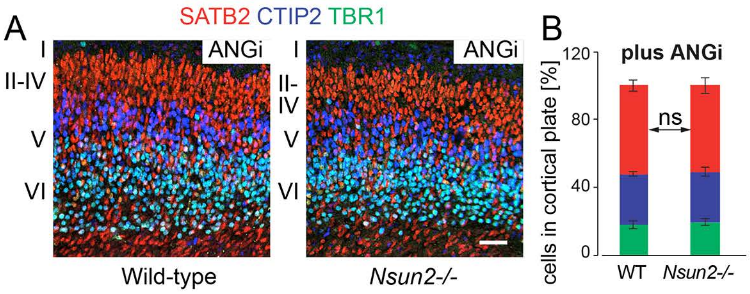

Supplementary Figure 1. (A) Cortical section of wild-type (WT) and *Nsun2*-/-^{D014D11} mouse brains at E18.5 treated with angiogenin inhibitor (Angi). The layers are labeled for markers for layer VI (TBR1), layer V (CTIP2) and layer IV (SATB2). (B) Quantification of (A) showing no statistically significant difference in populations in the cortical plate in wild-type versus *Nsun2*-/- brains. Data represent mean ± sd. (n=3 mice per genotype). Significance was assessed using student's t-test. Ns: Not significant. Scale bar: 50 μm.

Figure S2, Related to Figure 5

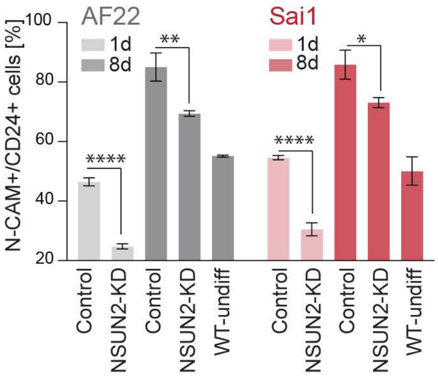

Supplementary Figure 2. Percentage of N-CAM and CD24 double-positive AF22 and Sai1 cells after 1 day (1d) and 8 days (8d) induction to differentiate (see Fig. 5E). The cells were transfected with scrambled control siRNA (control) or NSUN2 siRNAs (NSUN2-KD). As control undifferentiated wild-type cells for 8 days in culture were measured (WT-undiff). Data represent mean ± sd. (n=3 experiments per condition). Significance was assessed using student's t-test. **** $p<0.0001$; ** $p=0.0049$; * $p=0.0132$.
