# Supplementary material for: Cytosine-5 RNA Methylation Regulates Neural Stem Cell Differentiation and Motility
Source: Stem Cell Reports. 2016 Dec 29;8(1):112–24. doi: 10.1016/j.stemcr.2016.11.014 (PMC5233436; doi:10.1016/j.stemcr.2016.11.014)
Supplement: Document S2. Article plus Supplemental Information [file mmc7.pdf]

Stem Cell Reports

Article

OPEN ACCESS

Cytosine-5 RNA Methylation Regulates Neural Stem Cell Differentiation and Motility

Joana V. Flores,[1] Lucía Cordero-Espinoza,[1,2,5] Feride Oeztuerk-Winder,[1,3] Amanda Andersson-Rolf,[1] Tommaso Selmi,[1,3] Sandra Blanco,[1,3] Jignesh Tailor,[4] Sabine Dietmann,[1] and Michaela Frye[1,3,*]

[1]Wellcome Trust – Medical Research Council Cambridge Stem Cell Institute, University of Cambridge, Tennis Court Road, Cambridge CB2 1QR, UK
[2]Wellcome Trust/Cancer Research UK Gurdon Institute, Henry Wellcome Building of Cancer and Developmental Biology, Tennis Court Road, Cambridge CB2 1QN, UK
[3]Department of Genetics, University of Cambridge, Downing Street, Cambridge CB2 3EH, UK
[4]Department of Neurosurgery, King's College Hospital, Denmark Hill, London SE5 9RS, UK
[5]Department of Physiology, Development and Neuroscience, University of Cambridge, Cambridge CB2 3DY, UK
*Correspondence: mf364@cam.ac.uk
http://dx.doi.org/10.1016/j.stemcr.2016.11.014

SUMMARY

Loss-of-function mutations in the cytosine-5 RNA methylase NSUN2 cause neurodevelopmental disorders in humans, yet the underlying cellular processes leading to the symptoms that include microcephaly remain unclear. Here, we show that NSUN2 is expressed in early neuroepithelial progenitors of the developing human brain, and its expression is gradually reduced during differentiation of human neuroepithelial stem (NES) cells in vitro. In the developing *Nsun2*$^{-/-}$ mouse cerebral cortex, intermediate progenitors accumulate and upper-layer neurons decrease. Loss of NSUN2-mediated methylation of tRNA increases their endonucleolytic cleavage by angiogenin, and 5′ tRNA fragments accumulate in *Nsun2*$^{-/-}$ brains. Neural differentiation of NES cells is impaired by both *NSUN2* depletion and the presence of angiogenin. Since repression of NSUN2 also inhibited neural cell migration toward the chemoattractant fibroblast growth factor 2, we conclude that the impaired differentiation capacity in the absence of NSUN2 may be driven by the inability to efficiently respond to growth factors.

INTRODUCTION

Human brain development begins with the differentiation of neural progenitor cells in the third gestational week and extends through to adolescence (Stiles and Jernigan, 2010). The first cells committed to a neural fate appear during gastrulation in a single sheet of cells with epithelial features (Stern, 2005). These neuroepithelial stem (NES) cells then differentiate further into multiple types of cells, including neurons, astrocytes, and other glial cells (Temple, 2001). The brain is the most complex organ and its formation requires a tight control of lineage-specific differentiation pathways. To date many transcriptional networks have been identified as important regulators of lineage specification, yet little is known about the function of posttranscriptional regulation in the developing brain.

Gene expression is dynamically controlled through reversible chemical modifications in DNA and histones (Bannister and Kouzarides, 2011; Deaton and Bird, 2011). Covalent modifications are also commonly found in RNA, but their precise role in regulating gene expression and translation remains less well understood. However, RNA modifications are crucial for development and aberrant deposition of RNA modifications can lead to complex human diseases, including neurodevelopmental disorders and cancer (Frye and Blanco, 2016; Popis et al., 2016).

Many of the more than 100 known chemical modifications found in RNA have been described decades ago (Machnicka et al., 2013), but their potentially very broad roles in regulating RNA metabolism emerged only recently. Novel transcriptome-wide approaches revealed vital roles for pseudouridine, N^6-methyladenosine, N^1-methyladenosine, and 5-methylcytosine (m^5C) in posttranscriptional gene regulation (Carlile et al., 2014; Dominissini et al., 2012, 2016; Hussain et al., 2013a; Khoddami and Cairns, 2013; Lovejoy et al., 2014; Meyer et al., 2012; Schwartz et al., 2014).

Cytosine-5 methylation in RNA is mediated by a large protein family of conserved RNA:m^5C-methyltransferases (Motorin et al., 2010). NSUN2 is one member of this family and methylates the vast majority of tRNAs as well as a small number of other non-coding (ncRNAs) and coding RNAs (cRNAs) (Blanco et al., 2014; Hussain et al., 2013a; Khoddami and Cairns, 2013). Loss of NSUN2-mediated methylation of tRNAs increases their affinity to the endonuclease angiogenin, resulting in increased cleavage of tRNAs and accumulation of 5′ tRNA fragments (Blanco et al., 2014, 2016). The function of tRNA-derived ncRNA fragments is to repress global protein translation (Gebetsberger et al., 2012; Ivanov et al., 2011).

The correct deposition of m^5C into RNAs is essential for normal development. Loss-of-function mutations in the *NSUN2* gene in both mouse and human cause growth retardation and neurodevelopmental deficits including microcephaly, as well as defects in cognition and motor function (Blanco and Frye, 2014). In the developing mouse brain, expression of NSUN2 is highest in the cerebral cortex, hippocampus, and striatum, and all of these areas show

© 2017 The Authors.
This is an open access article under the CC BY license (http://creativecommons.org/licenses/by/4.0/).

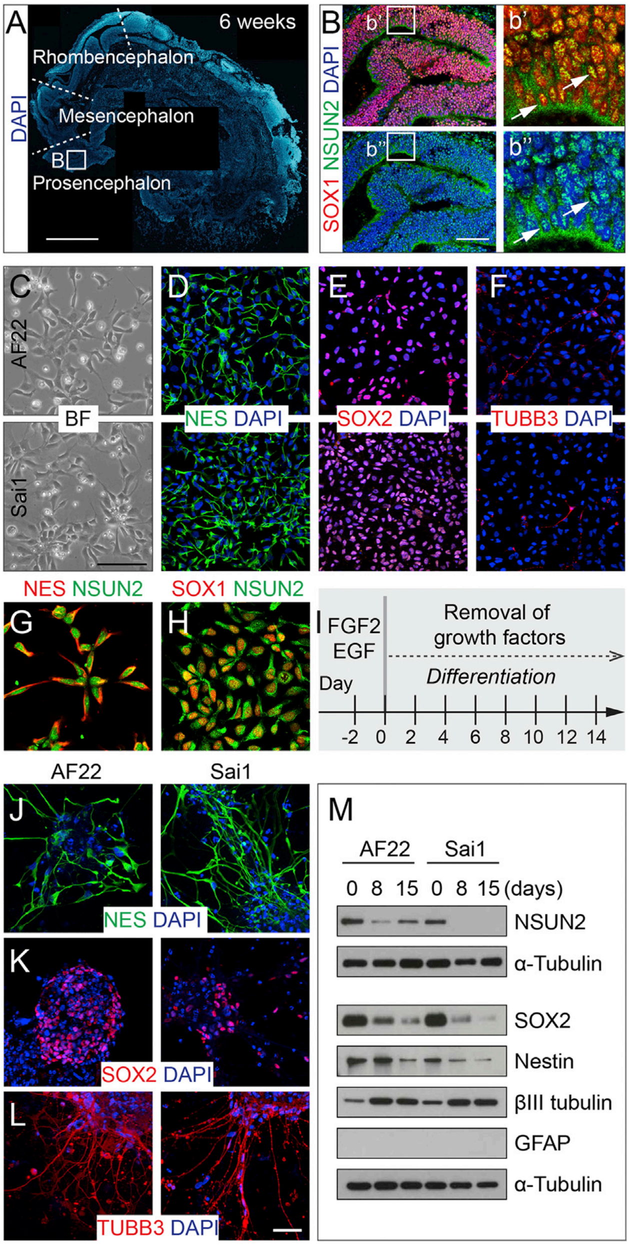

Figure 1. Expression of NSUN2 in the Human Developing Brain and NES Cells

(A) DAPI-stained human embryo (6 weeks of gestation) marked for prosencephalon, mesencephalon, and rhombencephalon. Region in square is magnified in (B). Scale bar, 1 mm.

(B) Prosencephalon labeled for NSUN2 and SOX1. Region in squares are magnified in (b′) and (b″). Arrows indicate NSUN2-positive cells. Scale bar, 100 μm.

(C–F) Bright-field image (C) and immunofluorescence (D–F) of AF22 (upper panels) and Sai1 (lower panels) cells labeled for Nestin (D), SOX2 (E), and βIII-tubulin (F). Scale bar, 50 μm.

(G and H) NES cells co-labeled for NSUN2 and Nestin (NES) (G) or SOX1 (H).

(I) Differentiation protocol.

(J–L) Differentiated AF22 and Sai1 cells (day 15) labeled for Nestin (NES; J), SOX2 (K), and βIII-tubulin (L). Scale bars: 50 μm.

(M) Western blot for NSUN2, βIII-tubulin (TUBB3), GFAP, SOX2, and Nestin during differentiation (days). α-Tubulin served as loading control.

Nuclei are counterstained with DAPI (A, B, D–F, J–L).

decreased global protein synthesis, increased cellular stress, and reduction in size in the absence of *Nsun2* (Blanco et al., 2014). Importantly, cleaved 5′ tRNA fragments are required and sufficient to induce the cellular stress responses, and both cellular stress and microcephaly can be rescued through inhibition of angiogenin (Blanco et al., 2014).

Here, we set out to dissect the underlying cellular process leading to the selective reduction in size of the cerebral cortex in the absence of NSUN2. In the developing mouse brain, deletion of *Nsun2* does not affect radial glia but delays differentiation into upper-layer neurons. In humans, NSUN2 is expressed in early neuroepithelial progenitors during development and cultured neuroepithelial stem/progenitor cells. Repression of NSUN2 is sufficient to inhibit neural migration and, in the presence of angiogenin, impairs neural lineage commitment. Thus, cytosine-5 RNA methylation pathways are required for the efficient cellular response toward neural lineage-inductive stimuli.

RESULTS

NSUN2 Is Expressed in Stem and Progenitor Cells during Human Brain Development

To detect NSUN2 in early human brain development, we performed immunohistochemistry on sagittal sections from 6-week-old embryos (Carnegie stage 16) (Figures 1A and 1B). Nucleolar expression of NSUN2 overlapped with SOX1, a marker for early neuroepithelial progenitors in the neural tube (Figures 1A and 1B). Thus, NSUN2 is expressed in early neuroectodermal cells that are capable of differentiating into various region-specific neuronal and glial cell types (Li et al., 2005; Perrier et al., 2004).

To characterize the expression of NSUN2 during human neural differentiation, we used an NES cell line (Sai1) isolated from embryonic hindbrain (Carnegie stage 15) and neuroepithelial-like stem cells (AF22) derived from pluripotent cells (Falk et al., 2012; Tailor et al., 2013). In proliferating conditions, AF22 and Sai1 cells showed the characteristic "rosette structures" (Figure 1C) (Wilson and Stice, 2006). Both lines expressed high levels of the NES cell markers Nestin and SOX2 but very low levels of the neural differentiation marker βIII-tubulin (TUBB3) (Figures 1D–1F). As expected, NSUN2 co-localized with Nestin and SOX1 in cultured NES cells (Figures 1G and 1H).

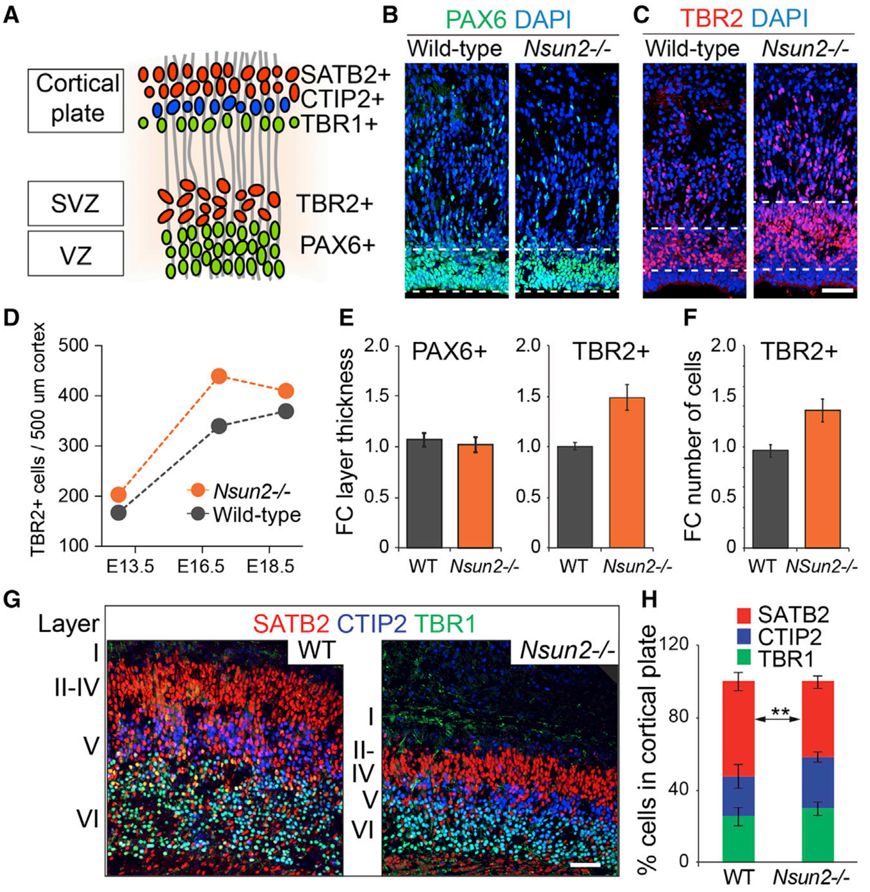

Figure 2. Reduction of Upper-Layer Neurons in the Nsun2$^{-/-}$ Cortex

(A) Mouse brain cortex at E18.5 showing the location of the indicated markers. Region in red rectangle is imaged in (B), (C), and (G).

(B and C) E18.5 mouse cortex labeled for PAX6 (B) and TBR2 (C) in wild-type (left-hand panels) and *Nsun2*$^{-/-}$ (right-hand panels) brains. The area between the dashed lines indicates marker-positive cells.

(D) Average of TBR2-positive (TBR2^{+}) cells in *Nsun2*$^{-/-}$ (MBKW) and wild-type littermates during development (n = 9 mice; five sections per data point).

(E and F) Increased thickness (E) and number (F) of TBR2^{+} cells was confirmed in an independent *Nsun2*$^{-/-}$ knockout line (D014D11). PAX6-positive (PAX6^{+}) cells were unaffected (E; left-hand panel). Data are mean ± SD (n = 3 sections from two mice per genotype). WT, wild-type.

(G) Cortical section of wild-type (WT) and *Nsun2*$^{-/-D014D11}$ mouse brains at E18.5 labeled for markers for layer VI (TBR1), layer V (CTIP2), and layer IV (SATB2).

(H) Quantification of (G) showing percentage of the indicated populations in the cortical plate. Data represent mean ± SD (n = 3 mice per genotype).

Significance was assessed using Student's t test. **p < 0.01. Scale bars, 50 μm. See also Figure S1.

Next, we induced differentiation of these cell lines by removal of the growth factors FGF2 (fibroblast growth factor 2) and EGF (epidermal growth factor) (Figure 1I). After 15 days in differentiation medium, the culture developed into complex multicellular aggregates with axonal-like growth at the periphery that still expressed Nestin and SOX2 but upregulated βIII-tubulin (Figures 1J–1L). In differentiation medium, we observed a gradual downregulation of NSUN2 together with Nestin and SOX2, βIII-tubulin expression was upregulated, and glial fibrillary acidic protein (GFAP), a marker for astrocytes, was not detectable (Figure 1M).

We concluded that human NSUN2 is expressed in stem and progenitor cells during early human brain development and downregulated during neural differentiation.

Upper-Layer Neurons Are Decreased in Nsun2 Knockout Brains

Loss-of-function mutations in *NSUN2* cause microcephaly in mouse and human (Blanco et al., 2014; Martinez et al., 2012). Therefore, we next asked whether the reduction in brain size might be due to an inadequate production of differentiated neurons during neurogenesis (Sun and Hevner, 2014). To visualize neuronal stem cells and their differentiated progeny, we labeled sections of the mouse cortex at embryonic day 18.5 (E18.5). At this stage of embryonic development NSUN2 is highly expressed in the cortex, hippocampus, and striatum, and the reduction of size of *Nsun2*$^{-/-}$ frontal brain lobe areas are most pronounced (Blanco et al., 2014).

In the brain cortex, stem cell populations (or radial glia) reside in the ventricular zone and express the transcription factor PAX6; intermediate progenitors localize to the subventricular zone and express TBR2 (Figure 2A) (Englund et al., 2005). While PAX6-positive layers were comparable, TBR2-positive layers increased in the absence of *Nsun2* (Figures 2B and 2C). The number of TBR2-positive cells was consistently higher in the *Nsun2*$^{-/-}$ cortex during embryonic development (Figure 2D). To confirm our findings, we measured the thickness of PAX6- and TBR2-postive layers in a second independent mouse *Nsun2* knockout line (*Nsun2*^{D014D11}). While similar in PAX6-positive layers, both layer thickness and number of TBR2-positive cells increased in the absence of NSUN2 (Figures 2E and 2F).

Next, we assessed the cortical plate, the area of the cortex to which mature neurons migrate during corticogenesis. Triple staining for TBR1, CTIP2, and SATB2 mark the neuronal layers VI, V, and IV–II, respectively (Figure 2A). Both the thickness and total number of cells in the cortical plate were reduced in *Nsun2*$^{-/-}$ brains (Figures 2G and 2H). However, only the percentage of the SATB2^{+} population was significantly reduced in the *Nsun2*$^{-/-}$ cortical plate (Figure 2H).

Thus, loss of *Nsun2* caused an accumulation of intermediate progenitors and a decrease in differentiated upper-layer neurons in the cortex.

Transfer RNAs Are Hypomethylated and Cleaved in Nsun2$^{-/-}$ Brains

To identify all methylation targets of NSUN2, we performed RNA bisulfite sequencing using the frontal brain regions from wild-type and *Nsun2*$^{-/-}$ mice at E18.5 (Tables S1, S2, and S3). The median conversion rate from cytosine to uracil was >99% in all RNA species and, as expected, tRNAs showed the overall lowest conversion rate (Figure 3A) (Blanco et al., 2014; Tuorto et al., 2012). Transcripts with >10% methylation in pooled wild-type samples showed on average significantly less methylation in the knockout brains (Figure 3B). However, methylation differences in coding RNA mostly occurred in transcripts with low coverage (Figure 3C) and, with the exception of tRNAs, the total number of m^{5}C sites with methylation levels >20% was low (Figures 3D–3F). The low coverage and level in methylation in cRNAs and ncRNAs might indicate that only a small fraction of transcripts carried NSUN2-dependent m^{5}C sites. Since we cannot exclude the possibility that non-tRNA RNAs contributed to the neurological defects in the mouse, we next asked whether tRNA hypomethylation was sufficient to explain the neural differentiation deficit observed in the absence of NSUN2.

Transfer RNAs lacking NSUN2-mediated methylation have a higher affinity to angiogenin (Blanco et al., 2014). Angiogenin cleaves tRNAs into small ncRNAs, causing a reduction in global protein synthesis (Figure 4A) (Blanco et al., 2016; Ivanov et al., 2011). To identify tRNA fragments in the absence of NSUN2, we performed small RNA sequencing using the frontal region of wild-type and *Nsun2*$^{-/-}$ brains at E13.5 and E18.5. We identified all differential abundant tRNA-derived small ncRNAs ($p < 0.01$) (Tables S4 and S5), and grouped them into 5′- and 3′-derived tRNA fragments (Figure 4B). We detected a significant enrichment of 5′-derived tRNA fragments over 3′-derived ncRNAs and other fragments (Figure 4C).

Thus, loss of NSUN2 increased cleavage of unmethylated tRNAs, leading to the enrichment of 5′-derived tRNA fragments during mouse brain development. To confirm that the cortical phenotype in *Nsun2*$^{-/-}$ mice (Figures 2A–2H) was caused by angiogenin-mediated cleavage of tRNAs, we injected pregnant *Nsun2*$^{+/-}$ females with the angiogenin inhibitor N65828 (Blanco et al., 2014; Kao et al., 2002). The reduction of the SATB2^{+} population in the cortical plate of *Nsun2*$^{-/-}$ mice (Figures 2G and 2H) was rescued by treatment with the angiogenin inhibitor (ANGi) (Figures S1A and S1B). Thus, we next asked whether angiogenin-mediated tRNA cleavage also affected human NES cell differentiation.

Angiogenin Reduces NES Cell Differentiation

NES cells were treated with recombinant angiogenin (rANG) and efficient incorporation of rANG was confirmed as early as 1 hr after exposure (Figures 4D and 4E). We also confirmed that even at high concentrations, rANG did not affect expression or nucleolar localization of NSUN2 (Figures 4F and 4G).

Next, we transfected undifferentiated NES cells with an *NSUN2* small interfering RNA (siRNA) and exposed the cells to differentiation medium (Figure 5A). We only measured a significant reduction of *TUBB3* RNA expression after 8 days of differentiation (Figures 5B and 5C). Treatment of rANG alone only slightly and insignificantly affected *TUBB3* RNA expression in the whole cell population (Figure 5D). Upon removal of EGF and FGF2, NES cells differentiate asynchronously into TUBB3-positive neurons, GFAP-positive and S100b-positive astroglial cells, and rarely into O4-positive cells indicative of the oligodendrocyte lineage (Tailor et al., 2013). To test a more homogeneous and pure population for neural differentiation, we used flow cytometry for the cell-surface markers CD24 and NCAM (CD56). In human cells, CD24^{high} populations occur with the transition from a mixed neural precursor cell population to the neuronal lineage, and a double-positive (CD24^{high}/CD56^{high}) population is consistent with differentiation toward neuroblasts and neurons (Pruszak et al., 2007, 2009; Schmandt et al., 2005).

To assess the differentiation potential of NES cells in the absence of NSUN2-methylated RNAs but in the presence of angiogenin, we simultaneously treated the cells with an siRNA for *NSUN2* and exposed them to rANG (Figure 5E). Both the NCAM^{+} population and the double-positive (NCAM^{+}/CD24^{+}) cell population increased with differentiation (Figures 5F and 5G). However, the proportion of double-positive cells was significantly reduced when exposed to the *NSUN2* siRNA or rANG (Figures 5F and 5G). The strongest inhibitory effect on differentiation occurred in cells treated with both the *NSUN2* siRNA and rANG (Figure 5G). These data were confirmed using a different set of *NSUN2* siRNAs (Figure S2). Together, these results suggest that low expression of NSUN2 and exposure to

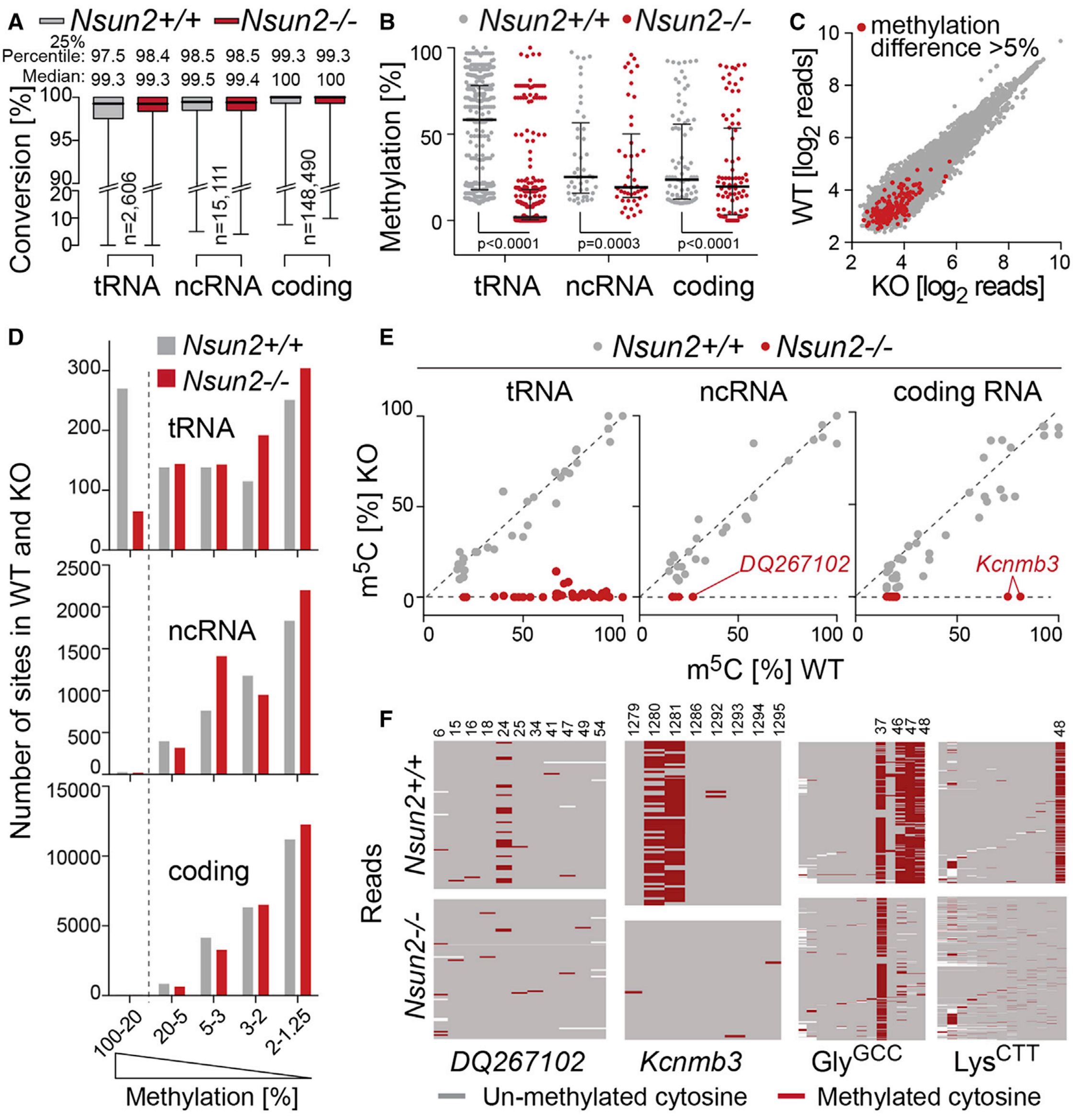

Figure 3. Loss of m^5C in RNA of Nsun2$^{-/-}$ Brains

(A) Bisulfite conversion rate (cytosine to uracil) in tRNA, non-coding (ncRNA), and coding RNA (cRNA) in wild-type (*Nsun2*$^{+/+}$) and knockout (*Nsun2*$^{-/-}$) brains at E18.5. Box plots show the median and interquartile range from minimum to maximum.

(B) Level of methylation in tRNA, ncRNA and cRNA in wild-type (*Nsun2*$^{+/+}$) and *Nsun2*$^{-/-}$ brains (sites with >10% methylation in pooled wild-type samples).

(C) Correlation of coverage in cRNAs from *Nsun2*$^{+/+}$ (WT) versus *Nsun2*$^{-/-}$ (KO) samples. Red dots indicate pooled methylation differences >5% (WT-KO).

(D) Number of m^5C sites in tRNA (upper panel), ncRNA (middle panel), and cRNA (lower panel) with the indicated level of methylation in *Nsun2*$^{+/+}$ (WT; gray) and *Nsun2*$^{-/-}$ (KO; red) brain samples.

(E) Correlation of methylation level in WT and KO samples (sites >10 reads coverage and >15% methylation in WT [mouse 1] versus KO [mouse 4]).

(F) Examples of NSUN2-targeted ncRNA (snoRNA *DQ267102*), cRNA (*Kcnmb3*), and tRNAs (Gly^{GCC} and Lys^{CTT}) in *Nsun2*$^{+/+}$ (upper panels) and *Nsun2*$^{-/-}$ (lower panels) brains represented as heatmaps. Red, methylated cytosines; gray, unmethylated cytosines; x axis, cytosines; y axis, reads.

Data are averaged/pooled from four mice per genotype (A–D, F).

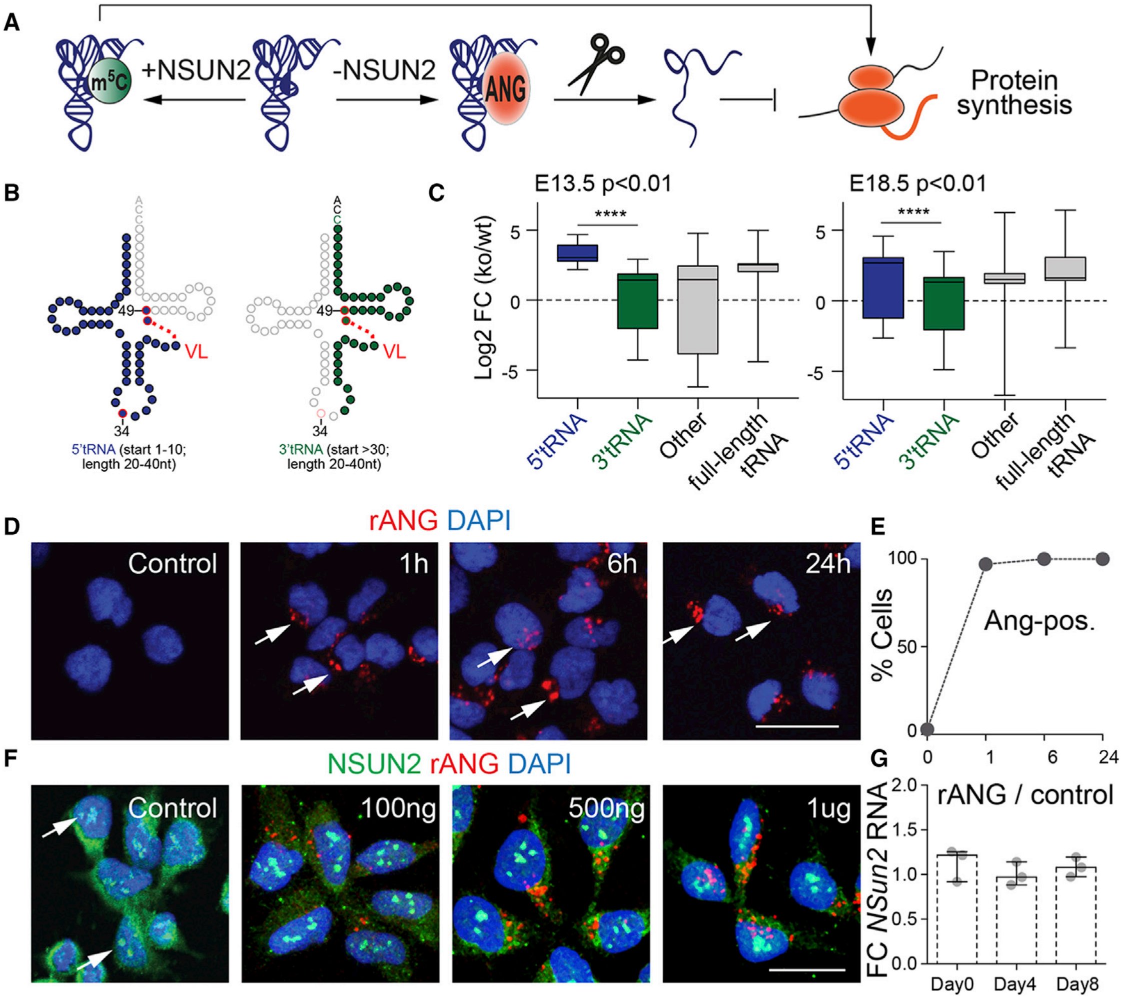

Figure 4. tRNA-Derived Small Non-coding RNAs Accumulate in Nsun2$^{-/-}$ Brains

(A) Angiogenin-mediated tRNA cleavage in the absence of NSUN2-dependent methylation.

(B) tRNA secondary structure highlighted for 5′-derived (blue) and 3′-derived (green) small ncRNAs (20–40 nucleotides). 5′ tRNA fragments start at position 1–10 and 3′ tRNA fragments start after position 30. VL, variable loop; red circle, methylated sites.

(C) Enrichment (log_2 fold change [FC]) of 5′-derived tRNA fragments (blue) compared with 3′-derived fragments (green), other fragments (start at position 11–30; any length <70), and full-length tRNAs (70–100) at E13.5 and E18.5. Box plots: n = 4 mice per genotype. ****$p < 0.0001$, Mann-Whitney U test.

(D) NES cells incubated with recombinant angiogenin (rANG) for the indicated time points. Arrows indicate angiogenin-positive. Scale bar, 25 μm

(E) Percentage of cells with internalized rANG per image field.

(F) Nucleolar localization (arrows) of NSUN2 in NES cells. Cells are counterstained with DAPI. Scale bar, 25 μm.

(G) Fold change (FC) of mean *NSUN2* RNA expression levels in the presence of rANG versus control-treated Sai1 cells at days 0, 4, and 8 after induction to differentiate (n = 3 experiments per time point).

angiogenin have an additive effect in delaying neural differentiation.

Delay of Neural Differentiation as a Consequence of Impaired Motility

CD56 is abundantly expressed in the developing and adult brain and plays a pivotal role in neurogenesis, neuronal migration, and neurite outgrowth (Edelman, 1986; Edelman and Jones, 1998; Grumet et al., 1982; Rutishauser et al., 1983). Since cortical development entails stem cell differentiation into neurons and glia as well as migration to form all functional layers, we asked whether impaired migration of *Nsun2*$^{-/-}$ cells may explain delayed differentiation into upper-layer neurons.

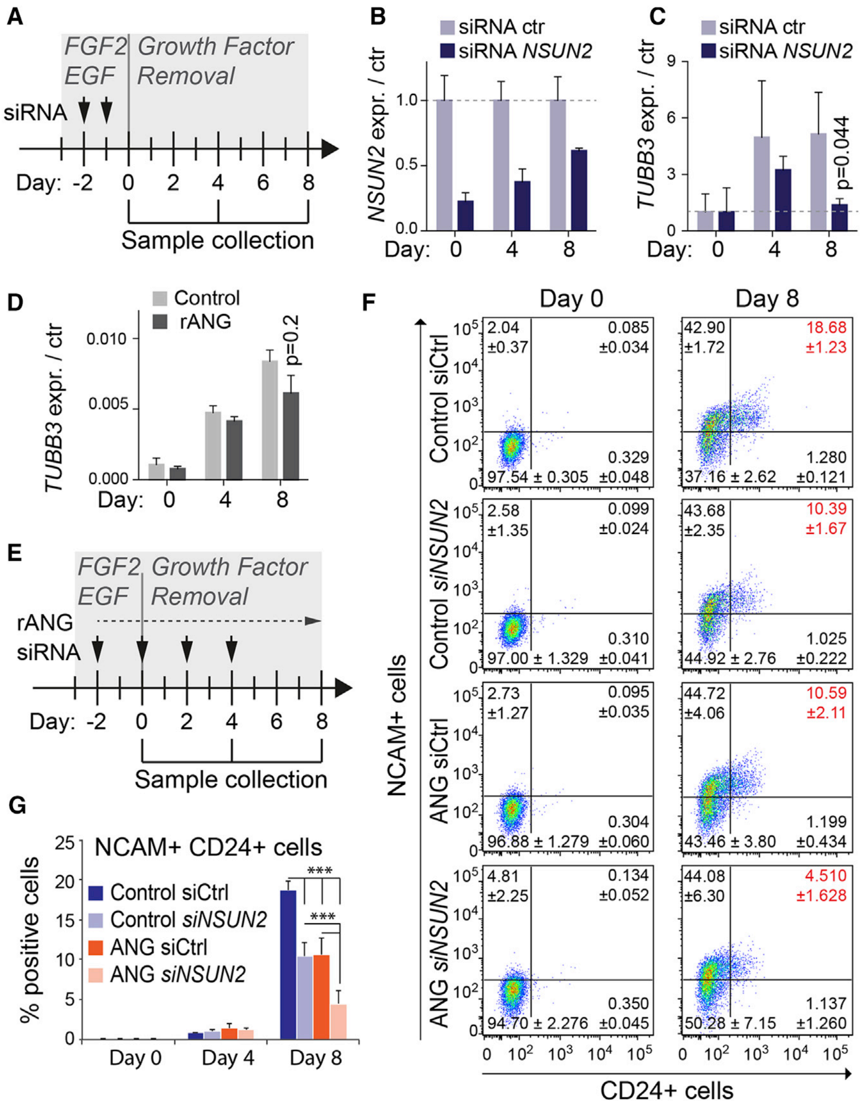

Figure 5. NSUN2 and Angiogenin Affect Neural Stem Cell Differentiation

(A) Transfection of siRNA (*NSUN2* and ctr), time course of NES cell differentiation, and sample collection.

(B–D) *NSUN2* (B) and *TUBB3* (C, D) RNA expression at days 0, 4, and 8 after growth factor removal in the presence of an *NSUN2* siRNA (B and C) or recombinant angiogenin (rANG) (D). Shown is ΔΔCt relative to GAPDH. Error bars denote SD (n = 3 transfections per time point).

(E) Treatment with rANG, the siRNAs for *NSUN2*, or a scrambled RNA as control (Ctrl), and time course of NES cell differentiation.

(F) Flow cytometry for NCAM and CD24 in control cells and cells treated with rANG and siRNAs (si*NSUN2* or siCtrl) at day 0 (left-hand panels) and day 8 (right-hand panels) of the differentiation protocol.

(G) Quantification of cells shown in (F). n = 5 experiments per condition. Error bars denote SD. ***$p < 0.001$, Student's t test.

See also Figure S2.

We transfected Sai1 and AF22 cells with siRNA constructs for *NSUN2* or scrambled RNA as control (Figure 6A) and plated 10,000 cells into Boyden chambers separated by a porous membrane from medium lacking or containing FGF2 as a chemoattractant (Figure 6B). *NSUN2*-silenced Sai1 and AF22 cells (siNSUN2) were less efficient in migration through the membrane compared with control cells, in particular toward medium containing FGF2 (Figures 6C and 6D). Thus, loss of *NSUN2* inhibited FGF2-induced cell migration of NES cells.

In summary, loss of NSUN2 impairs normal brain development by reducing the number of differentiated upper-layer neurons in the cortical plate. Since loss of NSUN2 also impaired cellular migrations, the delay in neural differentiation might rather be caused by the inability of *NSUN2*$^{-/-}$ cells to efficiently respond to lineage-inducing cytokines than by direct interference with the expression of differentiation genes (Figure 7).

DISCUSSION

Loss of function of NSUN2 impairs normal brain development and leads to microcephaly in mouse and human (Blanco et al., 2014; Martinez et al., 2012). Here, we reveal that the microcephaly phenotype can be explained by a reduced number of upper-layer neurons in the cerebral cortex. The precise timing of self-renewal and differentiation of neural progenitor cells as well as their migration and correct expansion are essential for normal mammalian brain development (Kriegstein and Alvarez-Buylla, 2009). Disturbance of any of these processes can lead to microcephaly (Manzini

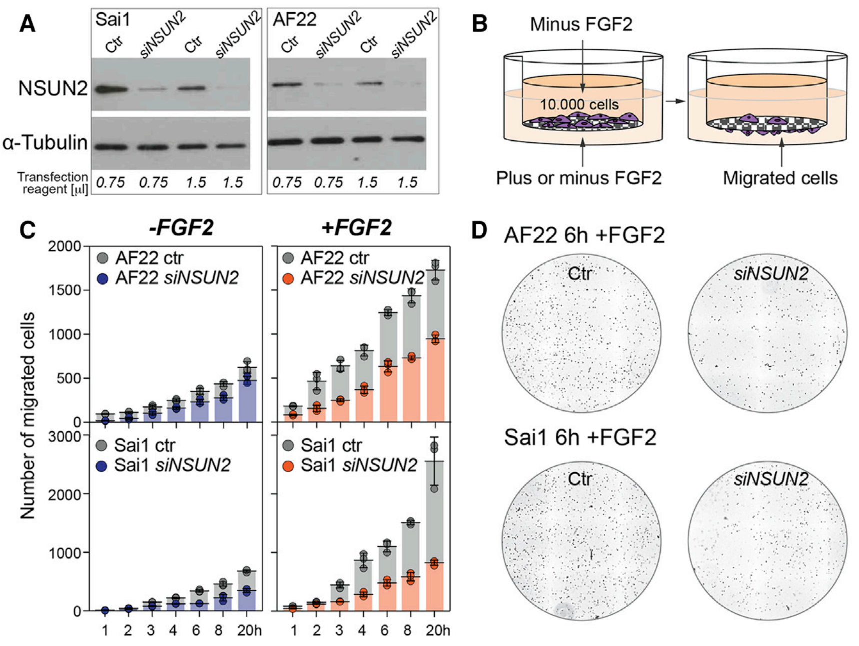

Figure 6. Reduced Migration of Neuroepithelial Stem Cells in the Absence of NSUN2

(A) Western blot for NSUN2 after transfecting Sai1 or AF22 cells with *NSUN2* or scrambled (Ctr) siRNAi constructs using two different amounts of transfection reagents (in µL).

(B) Boyden chamber assay to measure migration in the presence or absence of FGF2. A total of 10,000 cells were plated.

(C) Quantification of migrated cells at the indicated time points shown as mean ± SD (n = 3 wells per time point and condition).

(D) Example image of migrated cells in the presence of FGF2 after 6 hr. Nuclei are stained with DAPI. Ctr, cells transfected with scrambled RNAi.

and Walsh, 2011; Thornton and Woods, 2009). Similar to other tissues in the *Nsun2*$^{-/-}$ mice, the brain developmental deficits were not due to impaired cellular proliferation (Blanco et al., 2011; Hussain et al., 2013b). On the contrary, we found an increase in TBR2-positive intermediate progenitors in the *Nsun2*$^{-/-}$ cortex. The increase in progenitors but decrease of SATB2-positive upper-layer neurons strongly suggested a delay in differentiation.

To understand how NSUN2 and its methylated RNA targets interfere with neural differentiation pathways, we identified all NSUN2-dependent 5-methylcytosines in total RNA of the developing forebrain. In line with our previous studies in normal skin and skin tumors, we only find a robust loss of m^{5}C in tRNAs (Blanco et al., 2014, 2016). The overall lower methylation rate at distinct sites within cRNAs and ncRNAs might have several reasons: (1) the sites could be bisulfite conversion artifacts; (2) the sites are more difficult to detect due to the lower RNA stability compared with tRNAs; (3) the low percentage of methylation at distinct sites might indicate that only a small proportion of the expressed cellular RNA carried the modification. Thus, we cannot exclude that the few cRNAs and ncRNAs carrying NSUN2-dependent m^{5}C sites contribute to the brain developmental deficits. However, we now demonstrate that the impaired cellular differentiation is at least in part dependent on tRNA methylation. Differentiation of cultured human NES cells was less efficient in the absence of NSUN2, and the decreased capacity to differentiate was potentiated by angiogenin.

Angiogenin was the first human tumor-derived protein with in vivo angiogenic activity (Fett et al., 1985). Since then angiogenin has been implicated in a wide range of cellular responses including cell migration, invasion, proliferation, and formation of tubular structures (Gao and Xu, 2008). Small ncRNAs derived from angiogenin-mediated tRNA cleavage inhibit endothelial cell migration and tube formation (Li et al., 2016). Deletion of NSUN2 in induced tRNA cleavage, and reduced migration of human epidermal cells (Blanco et al., 2016).

Impaired neural migration and defects in the actin cytoskeleton are well-known factors contributing to malformations during the cortical development (Pang et al., 2008). Reduced expression of NSUN2 in human NES cells impaired migration toward the chemoattractant FGF2. FGF2 is widely used to maintain neural progenitors in long-term culture (Ostenfeld and Svendsen, 2004), is crucial for normal brain development, and is highly expressed in the ventricular zone when neural progenitors are formed (Dono et al., 1998; Vaccarino et al., 1999). Interestingly, injection of FGF2 into the mouse cerebral ventricles at E14 in utero did not affect the alignment of the cerebral neuronal layers; however, both cell number and cell density of the upper layers (II/III) and the lower layers (IV–VI) of the cerebral cortex were increased (Ohmiya et al., 2001).

In conclusion, our data support a model in which NSUN2-mediated tRNA methylation is required for efficient migration and differentiation of intermediate progenitors in the ventricular zone toward the upper-layer neurons. Failure to produce NSUN2 protein during development reduces the sensitivity toward growth factors and decreases the number of upper-layer neurons, and causes neurodevelopment deficits including microcephaly.

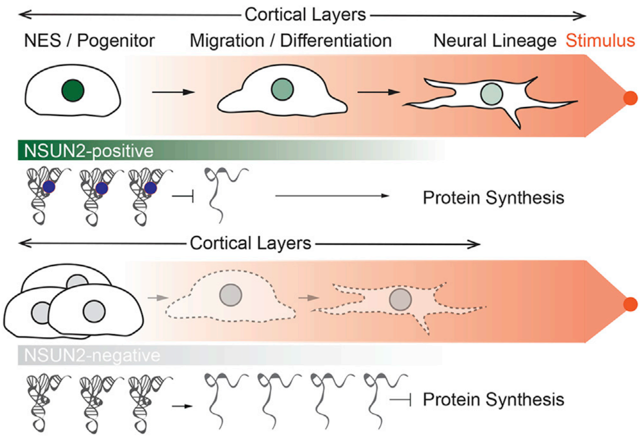

Figure 7. Summary of NSUN2 Functions in NES and Progenitor Cells

Shown are effects on tRNA methylation (blue dots), cell differentiation, and migration, as well as thickness of cortical layers in the presence (NSUN2-positive) and absence (NSUN2-negative) of NSUN2. Dotted lines show the reduction of migration and differentiation. Orange dot represents the chemoattractant.

EXPERIMENTAL PROCEDURES

Ethics Statements

All studies with human tissue were performed under ethical approval from the Cambridgeshire Research Ethics Committee (Reference 96/085) using tissue donated with informed consent after elective termination of pregnancy. The storage and use of human tissue were approved by the Human Tissue Authority, UK (License 12196).

The mice were housed in the Wellcome Trust - Medical Research Council Cambridge Stem Cell Institute Animal Unit. All mouse husbandry and experiments were carried out according to the local ethics committee under the terms of a UK Home Office license PPL80/2619 and PPL70/7822.

Transgenic Mice

Nsun2$^{-/-}$ mice (D014D11) and the MBKW mouse strain (*Nsun2*$^{tm1a(EUCOMM)Wtsi}$) are described in Blanco et al. (2011). The angiogenin small-molecule inhibitor N65828 (8-amino-5-(4′-hydroxybiphenyl-4-ylazo)naphthalene-2-sulfonate) was obtained from the National Cancer Institute (http://dtp.cancer.gov). For in vivo administration, N65828 (400 μg/mL in PBS [pH.7.4]) was subcutaneously injected every second day into pregnant *Nsun2*$^{+/-}$ female mice at a dose of 2.5 mg/kg. Pregnant mice were treated from E12.5 until E18.5 (Blanco et al., 2014).

Culture and Differentiation of NES Cells

Sai1 NES cells were derived from 6-week-old human embryos and AF22 neuroepithelial-like stem cells from human induced pluripotent stem cells (Falk et al., 2012; Tailor et al., 2013). The cell lines were kindly provided by Austin Smith. All cell lines were maintained in humidified incubators at 5% CO_2 and 37°C. AF22 and Sai1 were grown in DMEM/F12/Glutamax medium (Thermo Fisher Scientific) supplemented with B27 (1:50, Thermo Fisher Scientific), N2 (1:100, Thermo Fisher Scientific), human EGF (10 ng/mL, Thermo Fisher Scientific), and FGF2 (10 ng/mL, Thermo Fisher Scientific 13256-029). Cells were passaged at ratios no higher than 1:3 to avoid low cell density. Cell dissociation was achieved using trypsin, which was later neutralized with Defined Trypsin Inhibitor (Thermo Fisher Scientific) and washed thoroughly with fresh medium. All tissue culture flasks and plates were coated with poly-L-ornithine (0.1 mg/mL, Sigma) at 37°C for 30 min and with laminin (10 μg/mL, Sigma) at 4°C overnight prior to cell seeding. Half of the culture medium of the cells was replaced every day to prevent spontaneous differentiation.

For the differentiation experiments, cells were seeded at low or high confluency on day 0 with complete medium containing FGF2 and EGF. The next day, cells were washed twice with PBS and cultured with fresh medium lacking the FGF2 and EGF growth factors. Every 2 days, half of the volume of the culture medium was replaced. At least twice a week, laminin (10 μg/mL) was added to the medium.

siRNA Transfection of NES Cells

AF22 and Sai1 cells were transfected with a negative control siRNA (Qiagen) or *Nsun2* siRNA (SI02655548; Qiagen) at a final concentration of 20 nM. For confirmation of the *Nsun2* siRNA results, cells were transfected with a pool of different siRNA constructs: SI00300230, SI04205411, SI04224668, SI04308640, and SI04325874 (Qiagen). The transfection was performed using the DharmaFECT (Dharmacon) according to the manufacturer's protocol using 0.75 or 1.5 μL of DharmaFECT transfection reagent. The transfection solution was added to the cells for 6–12 hr, after which the medium was changed.

For knockdown of *Nsun2* in differentiated cells, NES cells were subjected to several cycles of transfection followed by 1 day of medium change and recovery. Transfections were performed at days −2, 0, 2, and 4 of the differentiation protocol. For instance, the first transfection was performed 24 hr after passaging (differentiation day −2) and the medium was changed the following day. One day later, the cells were retransfected and the medium was changed to differentiation medium (day 0). When samples were collected at day 4, the last transfection was at day 2 and when samples were collected at day 8, the last transfection was at day 4.

Western Blot and Real-Time qPCR Analyses

Cells were lysed in RIPA buffer (50 mM Tris-HCl [pH 7.4], 150 mM NaCl, 1% NP-40, 0.5% sodium deoxycholate, and 0.1% SDS). Immediately before use, one tablet of the COMPLETE Mini EDTA-free protease inhibitor (Roche) was added. Mouse brains were lysed by either tissue homogenization or sonication. Lysates were incubated on ice for 20 min and cleared from cell debris by centrifugation at 16,000 × *g* for 20 min. Protein lysates were quantified using the Coomassie Protein Assay Kit (Thermo Scientific). Proteins were transferred onto Hybond-C nitrocellulose membranes (GE Healthcare). Membranes were blocked for 1 hr with 5% skim milk in 0.1% TBS-T (Tris-buffered saline with 0.1% Tween 20) buffer and incubated overnight at 4°C with the primary antibody diluted in the same blocking solution (1:2,000 NSUN2 [hmetA]; 1:50,000 α-tubulin [ab4074; Abcam]; 1:400 TUBB31 [MAB1195; R&D

Systems]; 1:500 GFAB [M0761; Dako]; 1:1,000 Sox2 [ab97959; Abcam]; 1:1,000 Nestin [SC21248; Santa Cruz Biotechnology]). The following day the membranes were washed and incubated for 1 hr at room temperature with the appropriate horseradish peroxidase (HRP)-conjugated antibody diluted in the blocking buffer. Three more washes were performed prior to incubation with the ECL Prime Western Blotting Detection Reagent (GE Healthcare) for detection of HRP-mediated chemiluminescence.

Total RNA from cultured cells or mouse brains was extracted using TRIzol following the manufacturer's instructions (Thermo Fisher Scientific). The Superscript III Reverse Transcriptase kit (Thermo Fisher Scientific) was used to generate cDNA from total RNA, according to the manufacturer's instructions. Real-time PCR amplification and analysis was conducted using either the 7900HT Real-Time PCR System (Applied Biosystems) or the QuantStudio 12K Flex Real-Time PCR System (Applied Biosystems). The fast amplification protocol was performed with pre-designed probe sets (NSUN2: Hs00214829_m1; TUBB3/TUJ1: Hs00801390_s1) and TaqMan Fast Universal PCR Master Mix (2×) (Applied Biosystems). GAPDH was used as endogenous control (4352934E; Applied Biosystems).

Immunofluorescence of Tissue and Cells

Frozen gelatin/sucrose brain sections were incubated in PBS at 37°C for 30 min to remove gelatin. Antigen retrieval was performed by incubating the samples with boiling 0.01 M sodium citrate (pH 6.0) for 15 min, after which the samples were allowed to cool down and were washed twice with TBS. The slides were then incubated with blocking solution containing 5% donkey serum in TBS-T for 1 hr at room temperature. Primary antibodies were diluted in blocking solution as follows: 1:200 Sox1 (AF3369; R&D); 1:200 Nsun2 (hmetA); 1:500 Nestin MAB1259; R&D); 1:500 Sox2 (ab75485; Abcam); 1:400 TUBB3 (MAB1195; R&D); 1:400 Pax6 (AB2237; Millipore); 1:500 Tbr1 (ab31940; Abcam); 1:300 Tbr2 (ab23345; Abcam); 1:200 Ctip2 (ab18465; Abcam); 1:500 Satb2 (ab51502; Abcam). These antibodies were incubated overnight at 4°C. The slides were then washed three times with TBS and incubated with secondary antibodies diluted in the blocking solution (1:500) for 1 hr at room temperature. Tissues were washed thrice with TBS and incubated with DAPI (1:10,000) in PBS. Tissues were mounted onto slides using Mowiol.

Neuroepithelial cell lines were seeded onto glass coverslips coated with poly-L-ornithine and laminin. Cells were fixed with 4% paraformaldehyde (PFA) for 10 min, washed three times with PBS, and permeabilized with 0.2% Triton X-100 in PBS for 10 min, followed by a 1-hr incubation step with blocking buffer containing 5% donkey serum (Sigma) in PBS-T (PBS with 0.1% Tween). Primary antibodies diluted in the blocking buffer were then added to the cells for overnight incubation at 4°C. Cells were washed three times with PBS-T and then incubated for 1 hr at room temperature with blocking buffer containing fluorophore-conjugated secondary antibodies (1:500) and DAPI for 1 hr. After three more washes with PBS-T, the cells were mounted using Mowiol.

Image Acquisition and Quantification

All fluorescence images were acquired using a Leica SP5 confocal microscope. For quantifications, images of an area of 500 μm of cortex were taken. Bright-field images were acquired using an Olympus IX50 microscope. Quantifications were performed using Volocity software. Cortical marker quantifications were performed using Cell Profiler. All images were edited using ImageJ/Fiji.

Flow Cytometry

NES cells were grown in medium with or without growth factors and treated with siRNA for *NSUN2* and/or angiogenin (1 μg/mL; R&D, 265-AN-250/CF). Five biological replicates were collected for each condition. Cells were collected with Tryple Express and spun down. The cell pellet was washed and resuspended in 2% BSA in PBS. For cell differentiation analysis, antibody dilutions were prepared in 2% BSA in PBS. The following antibody dilutions were used: anti-NCAM (PE-conjugated, Santa Cruz, sc-106 PE, 1:25); anti-CD24 (fluorescein isothiocyanate [FITC]-conjugated, Becton Dickinson, 560992, 1:50). Cells were mixed with antibodies and incubated on ice for 30 min. After washing, the cells were resuspended in 500 μL of PBS for flow cytometry analysis. The flow cytometry analysis was performed in an LSRFortessa flow cytometer (BD). Controls for gating were unstained cells of the same condition (days 0, 4, and 8). Cells stained with each individual fluorophore (i.e., only PE-conjugated anti-NCAM; only FITC-conjugated anti-CD24) were used to compensate for overlap between the emission waves of the two fluorophores. The data were analyzed using FlowJo software (www.flowjo.com).

Cell Migration Assay

The transwell inserts (8 μm, BD Biosciences) were placed into 24-well plates and coated from both sides with polyornithine for 2 hr at 37°C and with laminin overnight at 4°C. The inserts were washed with PBS and placed inside a new 24-well plate containing 500 μL of medium/well with FGF2 (10 mg/mL). AF22 and Sai1 cells were trypsinized, counted, and resuspended in appropriate medium (no growth factors or FGF2); 10,000 cells were plated per insert. Cells were incubated in a time course from 1 to 20 hr, after which the inserts were washed once with PBS, fixed with 4% PFA for 10 min, and washed three more times with PBS. Cells were stained with DAPI. Non-migrated cells were scratched off from the upper side of the membrane with a cotton bud, with PBS washes in between. Migrated DAPI$^+$ cells on the bottom side of the membrane were imaged with a colonyscan microscope. Cells were then quantified with the software CellProfiler.

Generation of RNA Bisulfite-Converted and tRNA Sequencing Libraries

For RNA extraction from mouse embryonic brains (E18.5; four biological replicates per genotype) the brains were snap frozen in liquid N_2 directly after dissection, and kept on dry ice and homogenized in 1 mL of TRIzol. TRIzol was then mixed with 0.2 volume of chloroform (200 μL), centrifuged, and the upper phase was mixed with 0.5 volume of isopropanol (500 μL). The following steps were performed according to the manufacturer's instructions. Total RNA was treated with TURBO DNAse (Thermo Fisher Scientific) according to the manufacturer's instructions and subjected to an acidic phenol/chloroform extraction. The RNA was

resuspended in H_2O and treated with the RiboZero Magnetic Kit (Epicentre, MRZH11124) to remove rRNA. At least 2 μg of RNA was used for the bisulfite library preparation.

The RNA was bisulfite-converted as described previously (Blanco et al., 2014). About 2 μg of RNA was diluted in 70 μL of 40% sodium bisulfite (pH 5.0) and DNA protection buffer (Imprint DNA Modification Kit, Sigma). The reaction mixture was incubated in a thermocycler for four cycles of 5 min at 70°C followed by 1 hr at 60°C. The reaction mix was then loaded onto Micro Bio-spin6 chromatography columns (Bio-Rad) to remove salts from solution. Total RNA was desulfonated by adding an equal volume of 1 M Tris (pH 9.0) to the reaction mixture and incubating it at 37°C for 1 hr. The RNA was then purified by ethanol precipitation.

The bisulfite-converted RNA was assessed for quality and concentration using a Bioanalyzer 2100 RNA nanochip (Agilent). For the generation of bisulfite sequencing libraries, at least 120 ng of bisulfite-converted RNA was used. Bisulfite sequencing libraries were prepared using the TruSeq Small RNA Preparation Kit (Illumina), mostly according to the manufacturer's instructions with the exception of the size-selection step before hybridization (not performed due to sample fragmentation). Firstly, 20,30-cyclic phosphate and 50-hydroxyl termini produced during the bisulfite/desulfonation reaction were end-repaired using T4 PNK and Spermidine (New England Biolabs). RNA adapters suitable for Illumina sequencing were then ligated and the RNA was reverse-transcribed at 50°C for 1 hr with SuperScript III and 1 mM of each dNTP (SuperScript III cDNA synthesis kit, Thermo Fisher Scientific). The final step of library preparation was an 18-cycle PCR amplification, with no further size-selection steps.

The tRNA sequencing libraries were generated from E13.5 and E18.5 embryonic mouse brains. Four independent biological replicates per genotype were generated. Total RNA was extracted and treated with DNAse and Ribozero. The remaining RNA fraction was size-selected with the MirVana Isolation Kit (Invitrogen) for 20- to 200-base-paired RNAs, following the manufacturer's instructions. The RNAs were deaminoacylated in 0.1 M Tris-HCl (pH 9.0) and 1 mM EDTA for 30 min at 37°C. Small RNA libraries were prepared using TruSeq Small RNA Preparation Kit (Illumina), according to the manufacturer's instructions. After adapter ligation and reverse transcription steps the cDNA was subjected to 16 cycles of PCR amplification with Phusion DNA polymerase (Thermo Fisher Scientific) and size-selected (140–210 bp) before sequencing. All samples were multiplexed and sequenced in a HiSeq platform (Illumina).

Next-Generation Sequence Data Analyses

For all data analyses, FastQC was used for the initial assessment of the quality and basic processing of the reads (http://www.bioinformatics.babraham.ac.uk/projects/fastqc). Sequencing adapters were trimmed from the 5′ and the 3′ ends of the reads using *cutadapt* (v1.4.2; https://pypi.python.org/pypi/cutadapt/1.4.2).

RNA Bisulfite Sequencing Analysis

Sequences for ENSEMBL transcripts and tRNAs were extracted in FASTA format to determine RNA methylation levels in the mouse brain samples. All transcript isoforms were considered, and in addition the longest gene at full length including introns was retained as a representative sequence to identify RNA methylation sites in introns. Cs were converted to Ts in the reference transcript sequences, and in the processed bisulfite sequencing reads. Alignment of converted bisulfite sequencing reads against converted transcript sequences were performed using *bowtie* (version 1.1.1; bowtie-bio.sourceforge.net; options "*–m 500 –v 2 –a –best –strata*"). Following alignments, the reads that aligned in sense direction were obtained, and the original transcript sequences and reads were used to compile RNA methylation (C/(C + T)) levels considering only cytosines with at least 5× coverage. Heatmaps displaying either C or T in the aligned reads at each cytosine position were generated using custom PERL scripts and *matrix2png* (www.chibi.ubc.ca/matrix2png/) for visualization. Cytosine positions on the heatmaps were reported relative to the annotated transcriptional start sites of the transcripts.

The conversion rate of unmethylated cytosines to uracil in tRNAs, ncRNA, and coding RNA is the subtraction of the pooled methylation value from 100. Only sites that were covered by a minimum of 10 reads (average over four replicates) in wild-type and *Nsun2*$^{-/-}$ brains were included in the analysis. The pooled methylation value of each cytosine was estimated using four biological replicates per genotype.

tRNA Sequencing Data Analysis

The abundance of tRNA fragments was determined as described previously (Blanco et al., 2014). Adapter-trimmed tRNA sequencing reads (>20 nt and <200 nt in length) were mapped to the mouse reference genome (GRCm38/mm10) using *bowtie* (version 1.1.1; bowtie-bio.sourceforge.net; options "*-m1 –v2 –a –best –strata*") considering only reads that map uniquely to the genome. To account for the polymerization of CCA 3′ ends onto mature tRNAs, we trimmed the remaining unmapped reads of CCA[CCA] ends and realigned using the same options. Annotations were conducted based on tRNA genes predicted for the mouse reference genome (GRCm38/mm10) and downloaded from GtRNAdb (http://lowelab.ucsc.edu/GtRNAdb). Reads that exceeded the annotated tRNA gene start or end by more than 10% were discarded. All distinct reads, which were shorter than 90% of the annotated tRNA gene length, were considered as tRNA fragments.

Statistical Analyses

Unless indicated otherwise, the statistical analysis was assessed using an unpaired Student's t test using a minimum of three replicates per condition.

ACCESSION NUMBERS

The accession number for the sequencing data reported in this paper is GEO: GSE82162.

SUPPLEMENTAL INFORMATION

Supplemental Information includes two figures and five tables and can be found with this article online at http://dx.doi.org/10.1016/j.stemcr.2016.11.014.

AUTHOR CONTRIBUTIONS

J.V.F., L.C.-E, F.O.-W., A.A.-R., T.S., and S.B. performed experiments; J.T. provided cells and reagents; S.D. performed bioinformatics analyses; M.F. analysed data and wrote the paper.

ACKNOWLEDGMENTS

We thank everybody who provided us with reagents, in particular Austin Smith for providing the neuroepithelial stem cell lines and Anna Falk for advice on their culture. This work was funded by Cancer Research UK (CR-UK C10701/A15181), Worldwide Cancer Research (15-0168), the Medical Research Council (MRC MR/M01939X/1), the European Research Council (ERC 310360), and EMBO. Research in M.F.'s laboratory was supported by a core support grant from the Wellcome Trust and MRC to the Wellcome Trust-Medical Research Cambridge Stem Cell Institute.

Received: June 9, 2016
Revised: November 29, 2016
Accepted: November 30, 2016
Published: December 29, 2016

REFERENCES

Bannister, A.J., and Kouzarides, T. (2011). Regulation of chromatin by histone modifications. Cell Res. *21*, 381–395.

Blanco, S., and Frye, M. (2014). Role of RNA methyltransferases in tissue renewal and pathology. Curr. Opin. Cell Biol. *31C*, 1–7.

Blanco, S., Kurowski, A., Nichols, J., Watt, F.M., Benitah, S.A., and Frye, M. (2011). The RNA-methyltransferase Misu (NSun2) poises epidermal stem cells to differentiate. PLoS Genet. *7*, e1002403.

Blanco, S., Dietmann, S., Flores, J.V., Hussain, S., Kutter, C., Humphreys, P., Lukk, M., Lombard, P., Treps, L., Popis, M., et al. (2014). Aberrant methylation of tRNAs links cellular stress to neuro-developmental disorders. EMBO J. *33*, 2020–2039.

Blanco, S., Bandiera, R., Popis, M., Hussain, S., Lombard, P., Aleksic, J., Sajini, A., Tanna, H., Cortes-Garrido, R., Gkatza, N., et al. (2016). Stem cell function and stress response are controlled by protein synthesis. Nature *534*, 335–340.

Carlile, T.M., Rojas-Duran, M.F., Zinshteyn, B., Shin, H., Bartoli, K.M., and Gilbert, W.V. (2014). Pseudouridine profiling reveals regulated mRNA pseudouridylation in yeast and human cells. Nature *515*, 143–146.

Deaton, A.M., and Bird, A. (2011). CpG islands and the regulation of transcription. Genes Dev. *25*, 1010–1022.

Dominissini, D., Moshitch-Moshkovitz, S., Schwartz, S., Salmon-Divon, M., Ungar, L., Osenberg, S., Cesarkas, K., Jacob-Hirsch, J., Amariglio, N., Kupiec, M., et al. (2012). Topology of the human and mouse m6A RNA methylomes revealed by m6A-seq. Nature *485*, 201–206.

Dominissini, D., Nachtergaele, S., Moshitch-Moshkovitz, S., Peer, E., Kol, N., Ben-Haim, M.S., Dai, Q., Di Segni, A., Salmon-Divon, M., Clark, W.C., et al. (2016). The dynamic N(1)-methyladenosine methylome in eukaryotic messenger RNA. Nature *530*, 441–446.

Dono, R., Texido, G., Dussel, R., Ehmke, H., and Zeller, R. (1998). Impaired cerebral cortex development and blood pressure regulation in FGF-2-deficient mice. EMBO J. *17*, 4213–4225.

Edelman, G.M. (1986). Cell adhesion molecules in neural histogenesis. Annu. Rev. Physiol. *48*, 417–430.

Edelman, G.M., and Jones, F.S. (1998). Gene regulation of cell adhesion: a key step in neural morphogenesis. Brain Res. Brain Res. Rev. *26*, 337–352.

Englund, C., Fink, A., Lau, C., Pham, D., Daza, R.A., Bulfone, A., Kowalczyk, T., and Hevner, R.F. (2005). Pax6, Tbr2, and Tbr1 are expressed sequentially by radial glia, intermediate progenitor cells, and postmitotic neurons in developing neocortex. J. Neurosci. *25*, 247–251.

Falk, A., Koch, P., Kesavan, J., Takashima, Y., Ladewig, J., Alexander, M., Wiskow, O., Tailor, J., Trotter, M., Pollard, S., et al. (2012). Capture of neuroepithelial-like stem cells from pluripotent stem cells provides a versatile system for in vitro production of human neurons. PLoS One *7*, e29597.

Fett, J.W., Strydom, D.J., Lobb, R.R., Alderman, E.M., Bethune, J.L., Riordan, J.F., and Vallee, B.L. (1985). Isolation and characterization of angiogenin, an angiogenic protein from human carcinoma cells. Biochemistry *24*, 5480–5486.

Frye, M., and Blanco, S. (2016). Post-transcriptional modifications in development and stem cells. Development *143*, 3871–3881.

Gao, X., and Xu, Z. (2008). Mechanisms of action of angiogenin. Acta Biochim. Biophys. Sinica *40*, 619–624.

Gebetsberger, J., Zywicki, M., Kunzi, A., and Polacek, N. (2012). tRNA-derived fragments target the ribosome and function as regulatory non-coding RNA in *Haloferax volcanii*. Archaea *2012*, 260909.

Grumet, M., Rutishauser, U., and Edelman, G.M. (1982). Neural cell adhesion molecule is on embryonic muscle cells and mediates adhesion to nerve cells in vitro. Nature *295*, 693–695.

Hussain, S., Sajini, A.A., Blanco, S., Dietmann, S., Lombard, P., Sugimoto, Y., Paramor, M., Gleeson, J.G., Odom, D.T., Ule, J., et al. (2013a). NSun2-mediated cytosine-5 methylation of vault noncoding RNA determines its processing into regulatory small RNAs. Cell Rep. *4*, 255–261.

Hussain, S., Tuorto, F., Menon, S., Blanco, S., Cox, C., Flores, J.V., Watt, S., Kudo, N.R., Lyko, F., and Frye, M. (2013b). The mouse cytosine-5 RNA methyltransferase NSun2 is a component of the chromatoid body and required for testis differentiation. Mol. Cell Biol. *33*, 1561–1570.

Ivanov, P., Emara, M.M., Villen, J., Gygi, S.P., and Anderson, P. (2011). Angiogenin-induced tRNA fragments inhibit translation initiation. Mol. Cell *43*, 613–623.

Kao, R.Y., Jenkins, J.L., Olson, K.A., Key, M.E., Fett, J.W., and Shapiro, R. (2002). A small-molecule inhibitor of the ribonucleolytic activity of human angiogenin that possesses antitumor activity. Proc. Natl. Acad. Sci. USA *99*, 10066–10071.

Khoddami, V., and Cairns, B.R. (2013). Identification of direct targets and modified bases of RNA cytosine methyltransferases. Nat. Biotechnol. *31*, 458–464.

Kriegstein, A., and Alvarez-Buylla, A. (2009). The glial nature of embryonic and adult neural stem cells. Annu. Rev. Neurosci. *32*, 149–184.

Li, X.J., Du, Z.W., Zarnowska, E.D., Pankratz, M., Hansen, L.O., Pearce, R.A., and Zhang, S.C. (2005). Specification of motoneurons from human embryonic stem cells. Nat. Biotechnol. *23*, 215–221.

Li, Q., Hu, B., Hu, G.W., Chen, C.Y., Niu, X., Liu, J., Zhou, S.M., Zhang, C.Q., Wang, Y., and Deng, Z.F. (2016). tRNA-derived small non-coding RNAs in response to ischemia inhibit angiogenesis. Sci. Rep. *6*, 20850.

Lovejoy, A.F., Riordan, D.P., and Brown, P.O. (2014). Transcriptome-wide mapping of pseudouridines: pseudouridine synthases modify specific mRNAs in S. cerevisiae. PLoS One *9*, e110799.

Machnicka, M.A., Milanowska, K., Osman Oglou, O., Purta, E., Kurkowska, M., Olchowik, A., Januszewski, W., Kalinowski, S., Dunin-Horkawicz, S., Rother, K.M., et al. (2013). MODOMICS: a database of RNA modification pathways—2013 update. Nucleic Acids Res. *41*, D262–D267.

Manzini, M.C., and Walsh, C.A. (2011). What disorders of cortical development tell us about the cortex: one plus one does not always make two. Curr. Opin. Genet. Dev. *21*, 333–339.

Martinez, F.J., Lee, J.H., Lee, J.E., Blanco, S., Nickerson, E., Gabriel, S., Frye, M., Al-Gazali, L., and Gleeson, J.G. (2012). Whole exome sequencing identifies a splicing mutation in NSUN2 as a cause of a Dubowitz-like syndrome. J. Med. Genet. *49*, 380–385.

Meyer, K.D., Saletore, Y., Zumbo, P., Elemento, O., Mason, C.E., and Jaffrey, S.R. (2012). Comprehensive analysis of mRNA methylation reveals enrichment in 3′ UTRs and near stop codons. Cell *149*, 1635–1646.

Motorin, Y., Lyko, F., and Helm, M. (2010). 5-methylcytosine in RNA: detection, enzymatic formation and biological functions. Nucleic Acids Res. *38*, 1415–1430.

Ohmiya, M., Fukumitsu, H., Nitta, A., Nomoto, H., Furukawa, Y., and Furukawa, S. (2001). Administration of FGF-2 to embryonic mouse brain induces hydrocephalic brain morphology and aberrant differentiation of neurons in the postnatal cerebral cortex. J. Neurosci. Res. *65*, 228–235.

Ostenfeld, T., and Svendsen, C.N. (2004). Requirement for neurogenesis to proceed through the division of neuronal progenitors following differentiation of epidermal growth factor and fibroblast growth factor-2-responsive human neural stem cells. Stem Cells *22*, 798–811.

Pang, T., Atefy, R., and Sheen, V. (2008). Malformations of cortical development. Neurologist *14*, 181–191.

Perrier, A.L., Tabar, V., Barberi, T., Rubio, M.E., Bruses, J., Topf, N., Harrison, N.L., and Studer, L. (2004). Derivation of midbrain dopamine neurons from human embryonic stem cells. Proc. Natl. Acad. Sci. USA *101*, 12543–12548.

Popis, M.C., Blanco, S., and Frye, M. (2016). Posttranscriptional methylation of transfer and ribosomal RNA in stress response pathways, cell differentiation, and cancer. Curr. Opin. Oncol. *28*, 65–71.

Pruszak, J., Sonntag, K.C., Aung, M.H., Sanchez-Pernaute, R., and Isacson, O. (2007). Markers and methods for cell sorting of human embryonic stem cell-derived neural cell populations. Stem Cells *25*, 2257–2268.

Pruszak, J., Ludwig, W., Blak, A., Alavian, K., and Isacson, O. (2009). CD15, CD24, and CD29 define a surface biomarker code for neural lineage differentiation of stem cells. Stem Cells *27*, 2928–2940.

Rutishauser, U., Grumet, M., and Edelman, G.M. (1983). Neural cell adhesion molecule mediates initial interactions between spinal cord neurons and muscle cells in culture. J. Cell Biol. *97*, 145–152.

Schmandt, T., Meents, E., Gossrau, G., Gornik, V., Okabe, S., and Brustle, O. (2005). High-purity lineage selection of embryonic stem cell-derived neurons. Stem Cells Dev. *14*, 55–64.

Schwartz, S., Bernstein, D.A., Mumbach, M.R., Jovanovic, M., Herbst, R.H., Leon-Ricardo, B.X., Engreitz, J.M., Guttman, M., Satija, R., Lander, E.S., et al. (2014). Transcriptome-wide mapping reveals widespread dynamic-regulated pseudouridylation of ncRNA and mRNA. Cell *159*, 148–162.

Stern, C.D. (2005). Neural induction: old problem, new findings, yet more questions. Development *132*, 2007–2021.

Stiles, J., and Jernigan, T.L. (2010). The basics of brain development. Neuropsychol. Rev. *20*, 327–348.

Sun, T., and Hevner, R.F. (2014). Growth and folding of the mammalian cerebral cortex: from molecules to malformations. Nat. Rev. Neurosci. *15*, 217–232.

Tailor, J., Kittappa, R., Leto, K., Gates, M., Borel, M., Paulsen, O., Spitzer, S., Karadottir, R.T., Rossi, F., Falk, A., et al. (2013). Stem cells expanded from the human embryonic hindbrain stably retain regional specification and high neurogenic potency. J. Neurosci. *33*, 12407–12422.

Temple, S. (2001). The development of neural stem cells. Nature *414*, 112–117.

Thornton, G.K., and Woods, C.G. (2009). Primary microcephaly: do all roads lead to Rome? Trends Genet. *25*, 501–510.

Tuorto, F., Liebers, R., Musch, T., Schaefer, M., Hofmann, S., Kellner, S., Frye, M., Helm, M., Stoecklin, G., and Lyko, F. (2012). RNA cytosine methylation by Dnmt2 and NSun2 promotes tRNA stability and protein synthesis. Nat. Struct. Mol. Biol. *19*, 900–905.

Vaccarino, F.M., Schwartz, M.L., Raballo, R., Nilsen, J., Rhee, J., Zhou, M., Doetschman, T., Coffin, J.D., Wyland, J.J., and Hung, Y.T. (1999). Changes in cerebral cortex size are governed by fibroblast growth factor during embryogenesis. Nat. Neurosci. *2*, 246–253.

Wilson, P.G., and Stice, S.S. (2006). Development and differentiation of neural rosettes derived from human embryonic stem cells. Stem Cell Rev. *2*, 67–77.

Stem Cell Reports, Volume *8*

Supplemental Information

Cytosine-5 RNA Methylation Regulates Neural Stem Cell Differentiation and Motility

Joana V. Flores, Lucía Cordero-Espinoza, Feride Oeztuerk-Winder, Amanda Andersson-Rolf, Tommaso Selmi, Sandra Blanco, Jignesh Tailor, Sabine Dietmann, and Michaela Frye

Figure S1, Related to Figure 2

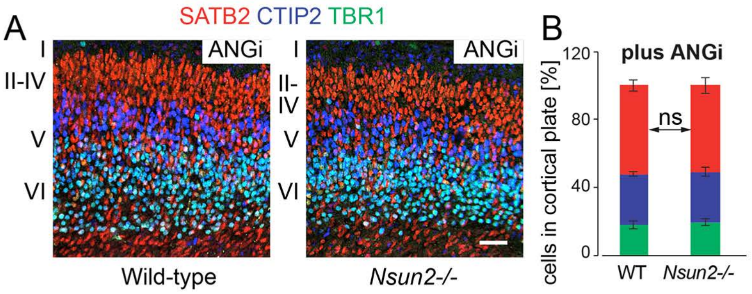

Supplementary Figure 1. (A) Cortical section of wild-type (WT) and *Nsun2*-/-^{D014D11} mouse brains at E18.5 treated with angiogenin inhibitor (Angi). The layers are labeled for markers for layer VI (TBR1), layer V (CTIP2) and layer IV (SATB2). (B) Quantification of (A) showing no statistically significant difference in populations in the cortical plate in wild-type versus *Nsun2*-/- brains. Data represent mean ± sd. (n=3 mice per genotype). Significance was assessed using student's t-test. Ns: Not significant. Scale bar: 50 μm.

Figure S2, Related to Figure 5

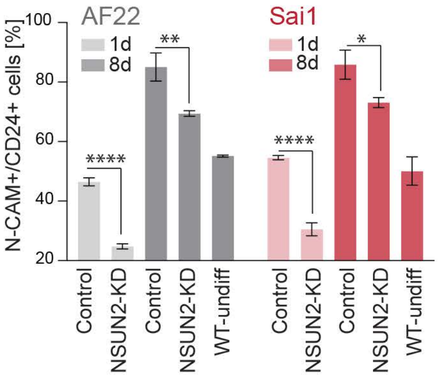

Supplementary Figure 2. Percentage of N-CAM and CD24 double-positive AF22 and Sai1 cells after 1 day (1d) and 8 days (8d) induction to differentiate (see Fig. 5E). The cells were transfected with scrambled control siRNA (control) or NSUN2 siRNAs (NSUN2-KD). As control undifferentiated wild-type cells for 8 days in culture were measured (WT-undiff). Data represent mean ± sd. (n=3 experiments per condition). Significance was assessed using student's t-test. **** $p<0.0001$; ** $p=0.0049$; * $p=0.0132$.
